# Optogenetic Stimulation of GABAergic Neurons in the Globus Pallidus Produces Hyperkinesia

**DOI:** 10.3389/fnbeh.2018.00185

**Published:** 2018-08-27

**Authors:** Jun Tian, Yaping Yan, Wang Xi, Rui Zhou, Huifang Lou, Shumin Duan, Jiang Fan Chen, Baorong Zhang

**Affiliations:** ^1^Second Affiliated Hospital, Zhejiang University School of Medicine, Hangzhou, China; ^2^Department of Neurobiology, School of Medicine, Zhejiang University, Hangzhou, China; ^3^School of Ophthalmology and Optometry, Wenzhou Medical University, Wenzhou, China

**Keywords:** optogenetic stimulation, GABAergic neurons, hyperkinesia, movement disorders, globus pallidus

## Abstract

The globus pallidus (GP) is emerging as a critical locus of basal ganglia control of motor activity, but the exact role of GABAergic GP neurons remain to be defined. By targeted expression of channelrhodopsin 2 (ChR2) in GABAergic neurons using the VGAT-ChR2-EYFP transgenic mice, we showed that optogenetic stimulation of GABAergic neurons in the right GP produced hyperkinesia. Optogenetic stimulation of GABAergic GP neurons increased c-Fos-positive cells in GP, M1 cortex, and caudate-putamen (CPu), and decreased c-Fos-positive cells in entopeduncular nucleus (EPN), compared to the contralateral hemisphere. In agreement with the canonical basal ganglia model. Furthermore, we delivered AAV-CaMKIIα-ChR2-mCherry virus to the excitatory neurons of the subthalamic nucleus (STN) and selectively stimulated glutamatergic afferent fibers from the STN onto the GP. This optogenetic stimulation produced abnormal movements, similar to the behaviors that observed in the VGAT-ChR2-EYFP transgenic mice. Meanwhile, we found that the c-Fos expression pattern in the GP, M1, STN, EPN, and CPu produced by optogenetic activation of glutamatergic afferent fibers from the STN in GP was similar to the c-Fos expression pattern in the VGAT-ChR2-EYFP transgenic mice. Taken together, our results suggest that excess GP GABAergic neurons activity could be the neural substrate of abnormal involuntary movements in hyperkinetic movement disorders. The neural circuitry underlying the abnormal involuntary movements is associated with excessive GP, M1, CPu activity, and reduced EPN activity. Inhibition of GP GABAergic neurons represents new treatment targets for hyperkinetic movement disorder.

## Introduction

The basal ganglia (BG), consisting of the striatum; the internal and external globus pallidus (GPi and GPe), which are also referred to as the globus pallidus (GP) and the entopeduncular nucleus (EPN) in rodents; the subthalamic nucleus (STN) and the substantianigra (SN), receives and processes cortical inputs and in return regulates cortical activity (Albin et al., 1989). The BG plays an important role in motor control through the direct and indirect pathways with the coordinated activity but often opposite effects on movement; the direct pathway selects specific motor programs/actions to facilitate movement, whereas the indirect pathway suppresses undesired motor programs (Albin et al., 1989; Chevalier and Deniau, 1990; Hikosaka, 2007; Gerfen and Surmeier, 2011; Rothwell, 2011). Consistent with the classical model of the basal ganglia, optogenetic activation of the striatal media spiny neurons (MSNs) in the direct pathway increases ambulation, while activation of the striatal MSNs in the indirect pathway decreases ambulation (Kravitz et al., 2010). The GP is traditionally considered as a homogeneous relay component of the “indirect pathway” (Albin et al., 1989; Gittis et al., 2014), but it is increasingly recognized as the central “hub” (Qiu et al., 2010, 2016; Goldberg and Bergman, 2011; Gittis et al., 2014) for its centrally placed structure in the basal ganglia that receives inputs from and projects to all major BG components (Grillner et al., 2005; Nambu, 2008; Goldberg and Bergman, 2011). The GP receives major GABAergic input through the afferent fibers from the striatopallidal projection neurons and glutamatergic input from the afferent fibers from the STN (Kita, 2007). The critical role of the GP in the control of movement is illustrated by the abnormal activity of GP neurons in movement disorders, including the increased firing rates in the GP in Huntington's disease (Starr et al., 2008) and beta oscillations of the GP neurons in Parkinson's disease (PD) (Mallet et al., 2008). Consistent with the critical role of the GP in basal ganglia circuits and behavior, quinolinic acid lesion of the GP leads to a decrease in spontaneous movement (Hauber et al., 1998) and activation of GP neurons by the microinjection of the GABAA receptor antagonist bicuculline (Matsumura et al., 1995) into the GP induces spontaneous movement (Grabli et al., 2004) and dyskinesia in primates (Crossman et al., 1984; Bronfeld et al., 2010).

However, the GP control of motor activity is complex since motor suppression has been associated with either electrophysiological pause (Kita and Kita, 2011), increased firing rate (Starr et al., 2008), or beta-oscillation (Pavlides et al., 2012). The firing rates of pallidal neurons are similar in Huntington's (with the increased abnormal movements) and PD patients (with reduced motor activity) (Tang et al., 2005). Studies with neurotoxin lesion and pharmacological activation have also produced results that are not consistent with the model. For example, GP lesion in rats increases (rather than decreases) spontaneous movement (Norton, 1976; Joel et al., 1998; Qiu et al., 2016). These mixed results, in part, reflect the complex neuronal connections of the GP with other structures of the basal ganglia and the molecular and functional diversity of GP neurons that contribute to motor and non-motor features of behavior (Mallet et al., 2012; Gittis et al., 2014; Mastro et al., 2014). The exact function of the GP in motor control largely remains to be defined.

The difficulty in defining GP function is, in part, due to technical limitations in selectively manipulating defined elements of the GP circuit in freely moving animals. The GP has heterogeneous neuronal populations; a majority of GP neurons (~95%) are GABAergic neurons, while a minority of GP neurons (~5%) are cholinergic neurons (Hegeman et al., 2016). GP GABAergic neurons can be divided into two classes based on their firing patterns, which are termed as “prototypical” neurons and “arkypallidal” neurons (Mallet et al., 2008; Abdi et al., 2015; Dodson et al., 2015). The prototypical and arkypallidal neurons project to distinct targets. The prototypical neurons project to STN and EPN, whereas arkypallidal neurons only project to the striatum (Mallet et al., 2012). The two populations of GABAergic GP neurons express different sets of transcription factors and have different roles (Abdi et al., 2015; Bahuguna et al., 2017; Lindahl and HellgrenKotaleski, 2017). A recent study found that activation of arkypallidal neurons suppressed motor output (Glajch et al., 2016). The exact role of GABAergic GP neurons in motor control is still unclear. Using the recently developed optogenetics (Boyden et al., 2005; Zhang et al., 2010) for manipulating the intrinsic GABAergic neurons in GP and glutamatergic afferents from STN in freely moving mice, we investigate the role of GABAergic GP neurons in the control of movement.

## Materials and methods

### Animals

The experiments were performed on 8- to 10-week-old mice. The VGAT-ChR2-EYFP mice were obtained from MinminLuo's laboratory at Beijing University, China (Zhao et al., 2011). In these mice, channelrhodopsin-2 (ChR2) was expressed in neurons under control of the vesicular GABA transporter (VGAT) promoter. VGAT was expressed by GABAergic neurons (Zhao et al., 2011) so that optical stimulation in these mice selectively stimulated GABAergic neurons. In addition, because the ChR2 was fused to EYFP, EYFP fluorescence was used to visualize the cellular localization of ChR2 (Henderson et al., 2014). All procedures were performed in accordance with the guidelines of the Zhejiang University Animal Experimentation Committee. This committee approved the experiments.

### Surgery

The mice were anesthetized with pentobarbital sodium (80 mg/kg, Sigma-Aldrich) and fixed to a stereotaxic apparatus (Stoelting). The guide cannula (RWD Life Science) was unilaterally implanted into the right GP [(Anterior–Posterior (AP): −0.3 mm, Medial–Lateral (ML): +1.9 mm, Dorsal-Ventral (DV): 2.6 mm) (according to the mouse brain in Stereotaxic Coordinates; Paxinos and Franklin, 2001)]. Four skull screw holes were drilled, and tightly fitting screws were driven through the skull until the surface of the dura was reached. Both the cannula and the stainless steel anchoring screws were fixed to the skull with dental cement. After the surgical procedures, the animals were allowed to recover in individual chambers for at least 7 days. During experimentation, each animal was transferred to a chamber and connected to an optical fiber.

### EEG recording

The animals were also implanted with a custom-made electroencephalogram (EEG) unit, which was placed on the rear of the skull, posterior to the site of cannula implantation. EEG signals were recorded from electrodes placed on the M1 cortex (AP: +2.1 mm, ML: +2.0 mm). The EEG signals from the implanted electrodes were monitored using the headstage of an EEG recording system (RM6240). Fast Fourier Transform (FFT) analyses were performed to determine the frequency spectrum.

### Virus injections

The AAV-CaMKIIα-ChR2-mCherry virus, which expressed ChR2 fused to the mCherry fluorescent protein under the control of the CaMKIIα promoter, was purchased from Shanghai SBO Medical Biotechnology. The virus was stereotaxically injected into eight C57/BL6 mice. The virus (0.3 μl) was injected into the right STN (AP: −1.8 mm, ML: +1.8 mm, DV: 3.6 mm) using Quintessential Stereotaxic Injector (Stoelting) at 40 nl/min. The mice were allowed to recover from the injection for 2 weeks for maximal virus expression prior to behavioral assessments.

### *In vivo* optical stimulation and behavioral tests

The optical fiber (0.2 mm in diameter) was inserted into the implanted cannula, and pulse trains of light (473 nm, 5–12 mW, 5 ms pulses at 20 Hz for 30 s) were delivered. The behavioral activities of the mice in response to optical stimulation were continuously observed and analyzed in the home cages. Eight types of behaviors were evaluated during the 30-s period that preceded optical stimulation, as well as during optical stimulation. The behaviors were defined as follows: (1) resting (awake without movements); (2) grooming; (3) exploration (moving in the cage); (4) licking; (5) chewing; (6) torsion spasms; (7) turning left; and (8) turning right. The duration of each behavior was quantified. Detailed behavioral analyses were performed offline using the recorded video. The analysis was made independently by two members of the laboratory who were blind to the treatments (none of the authors). Eight VGAT-ChR2-EYFP mice and eight mice infected with AAV-CaMKIIα-ChR2-mCherry were used to do the behavioral tests.

### Immunohistochemistry

To verify the targeted expression of the ChR2-expressing neurons in the GP and STN and their projections in the GP, the mice were anesthetized with pentobarbital sodium (160 mg/kg, Sigma-Aldrich) and transcardially perfused with physiological saline, followed by 4% paraformaldehyde. The brains were post-fixed in 4% paraformaldehyde overnight at 4°C. Coronal sections (30 μm) were treated with 0.3% Triton X-100 and placed in a PBS blocking solution containing 5% bovine serum albumin for 1 h at room temperature. The sections were then incubated with primary antibodies (mouse anti-GAD67, 1:1,000, millipore; goat anti-CaMKIIα, 1:500, abcam) in blocking solution for 1 day at 4°C. The sections were then washed in PBS three times for 5 min each and incubated for 2 h at room temperature with FITC-, Cy3-, or Cy5-conjugated secondary antibodies (1:1,000, SIGMA). To determine c-Fos immunoreactivity in the basal ganglia and cortex, the mice were continuously stimulated (5-ms pulses at 20 Hz) for 10 min and transcardially perfused 90 min later. After the sectioning and blocking procedures described above were completed, the sections were incubated with the primary antibody (rabbit anti-c-Fos, 1:500, calbiochem) for 1 day at 4°C. Then, the sections were washed in PBS three times for 5 min each and incubated for 2 h at room temperature with the Cy3-conjugated or Alexa 448-conjugated secondary antibody (1:1,000, SIGMA). The sections were then rinsed in 90% glycerol and coverslipped, and the immunostained neurons were analyzed. Ten VGAT-ChR2-EYFP mice, 10 wild-type mice, and eight mice infected with AAV-CaMKIIα-ChR2-mCherry were used for c-Fos immunohistochemistry. Three brain sections containing the GP (at −0.3 mm bregma), M1 (at 2.1 mm bregma), EPN (at −1.3 mm bregma), STN (at −1.8 mm bregma), and dorsal striatum (caudate-putamen, CPu) (at +0.3 mm bregma) per mouse were selected to count c-Fos-positive neurons. For each brain section, the total number of c-Fos-positive neurons in a given brain structure was counted and divided by the area occupied by this structure (in square millimeters) to obtain a density (number of c-Fos-positive neurons/area in square millimeters). c-Fos-positive neuron counts were performed at 20X magnification by an observer who was blind to the experimental treatment. Three sections for each area per mouse were analyzed. The resulting values were averaged for each area per mouse, and these averages were compared across groups.

### Statistical analysis

Behavioral studies were analyzed using a paired, two-tailed *t-*test. The paired, two-tailed *t-*test was performed both prior to and during optical stimulation. For c-Fos expression data, another paired, two-tailed *t-*test was performed between the sides ipsilateral and contralateral to the optical stimulation of mice transfected with AAV-CaMKIIa-ChR2 in STN. A two-way ANOVA, followed by *post-hoc* Tukey's test, was performed among three groups of VGAT-ChR2-EYFP transgenic mice and wild-type control mice. Statistical analysis was performed with SPSS version 17. *P* < 0.05 was taken to be statistically significant. The specific tests used were noted in the text and figure legends.

## Results

### Optical stimulation of GABaergic neurons in the right GP produced hyperkinesia

**Figure 1:**
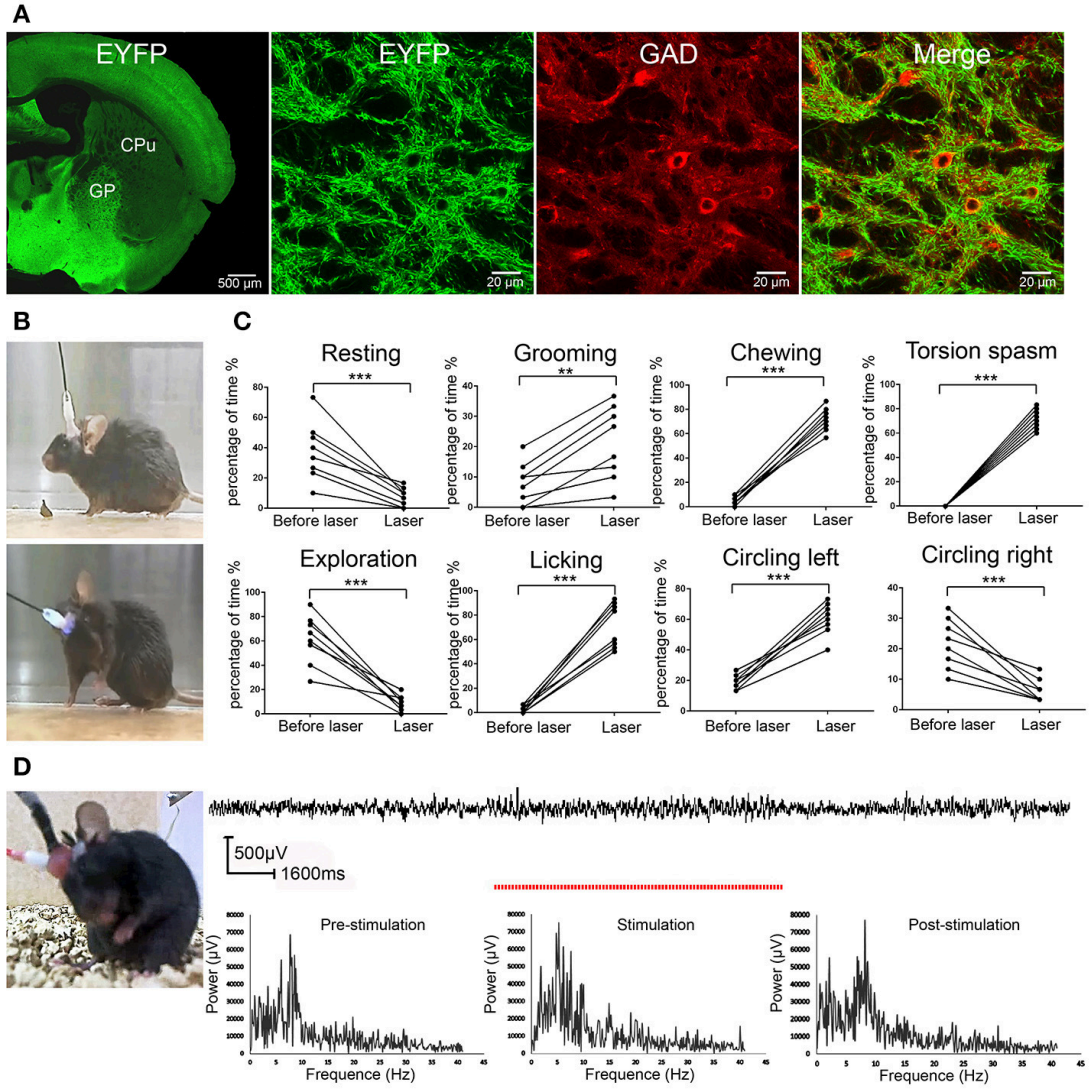
ChR2 activation of the GP GABAergic neurons induced dystonia-like behaviors in VGAT-ChR2-EYFP transgenic mice. **(A)** Specific expression of ChR2 in GP GABAergic neurons in VGAT-ChR2-EYFP transgenic mice. The confocal images show that GP GABAergic neurons expressed ChR2-EYFP and were co-stained with GAD67. **(B)** The mouse did not show any abnormal performance before light stimulation (upper panel) and showed torsion of the neck and forelimb after light stimulation of the right GP (lower panel). The symptoms could be seen more clearly in the video (Video S1). **(C)** The percentage of the 30-s period the animal spent engaging in each behavior. During optical stimulation of the right GP, the VGAT-ChR2-EYFP transgenic mice displayed more grooming, licking, chewing, dystonia-like behaviors (torsion of the neck and left forelimb) and circling left, and displayed less resting, exploration and circling right, compared to that prior to light stimulation [**p < 0.01, ***p < 0.001, *n* = 8, using a paired, two-tailed *t-*test; grooming: *t*_(7)_ = −4.989, *p* = 0.002; licking: *t*_(7)_ = −10.728, *p* < 0.001; chewing: *t*_(7)_ = −18.466, *p* < 0.001; torsion spasm: *t*_(7)_ = −24.826, *p* < 0.001; circling left: *t*_(7)_ = −10.458, *p* < 0.001; resting: *t*_(7)_ = 5.255, *p* = 0.001; exploration: *t*_(7)_ = 6.844, *p* < 0.001; circling right; *t*_(7)_ = 5.624, *p* = 0.001]. **(D)** EEG recording in the M1 cortex from a mouse that developed dystonia-like behaviors. The EEG signal in the M1 cortex did not show any sign of epileptic activity. The red dashed line represents the time of optical stimulation. Power spectrum of EEG signals recorded before, during, and after stimulus are depicted. There was no apparent increased power for the frequency range from 0.5 to 40 Hz during the stimulus.

We first verified that ChR2 expression was restricted to GABAergic neurons under control of VGAT promoter, which directed the selective expression of ChR2 in GABAergic and glycinergic neurons (Chaudhry et al., 1998) in the VGAT-ChR2-EYFP transgenic mice (Zhao et al., 2011). Consistent with the previous report (Zhao et al., 2011), we found that ChR2 was selectively expressed in GP GABAergic neurons as indicated by the co-localization of ChR2 and GAD67 (a marker for GABAergic neurons) in GP in the VGAT-ChR2-EYFP transgenic mice (Figure 1A). Thus, we employed the VGAT-ChR2-EYFP transgenic mouse line to manipulate the activity of GABAergic neurons for studying the behavioral response to light (ChR2) stimulation.

We applied 5-ms pulses of photostimulation at 20 Hz for 10–30 s to activate ChR2 in the GP GABAergic neurons and found that the mice developed dystonia-like, hyperkinetic motor symptoms (dystonia-like posture, repetitive grooming, licking, chewing, and circling left) in response to ChR2 stimulation (Figure 1B; Video S1). To quantify the abnormal behaviors produced by ChR2 stimulation, we adapted the following behavioral rating system: 1. resting (awake without movement); 2. grooming (cleaning its whiskers with its forelimbs); 3. exploration; 4. licking; 5. chewing; 6. torsion spasm; 7. circling left; and 8. circling right. The duration of the eight types of behavior was quantified in 30-s segments during ChR2 stimulation and over a 30-s control period before ChR2 stimulation. Under normal conditions, the mice primarily displayed resting, explorative, and grooming behaviors. During optical stimulation of the right GP, the VGAT-ChR2-EYFP transgenic mice displayed more stereotyped movements (grooming, licking, and chewing), as well as dystonia-like behaviors (torsion of the neck and left forelimb) and circling left, and displayed less resting, exploration and circling right, compared to that prior to light stimulation [^**^p < 0.01; ^***^p < 0.001; *n* = 8, using a paired, two-tailed *t-*test; grooming: *t*_(7)_ = −4.989, *p* = 0.002; licking: *t*_(7)_ = −10.728, *p* < 0.001; chewing: *t*_(7)_ = −18.466, *p* < 0.001; torsion spasm: *t*_(7)_ = −24.826, *p* < 0.001; circling left: *t*_(7)_ = −10.458, *p* < 0.001; resting: *t*_(7)_ = 5.255, *p* = 0.001; exploration: *t*_(7)_ = 6.844, *p* < 0.001; circling right; *t*_(7)_ = 5.624, *p* = 0.001] (Figure 1C). To exclude the possible epileptic effect of ChR2 stimulation in GP GABAergic neurons, we also simultaneously recorded EEGs from M1 cortex in the mice (*n* = 8) that developed dystonia-like behaviors under ChR2 stimulation. When the mice displayed dystonia-like behaviors, the EEG from M1 showed no sign of epileptic activities, indicating that the dystonia-like behaviors were not caused by seizures. We also performed FFT analyses on original EEG signals to determine the frequency spectra. There was no apparent increased power for the frequency range from 0.5 to 40 Hz during the stimulus. An example of EEG recordings is represented in Figure 1D.

### ChR2 stimulation of the GP GABaergic neurons produced the network level changes of the basal ganglia circuit, as indicated by c-Fos expression in the GP, M1, EPN, STN, and CPu

**Figure 2:**
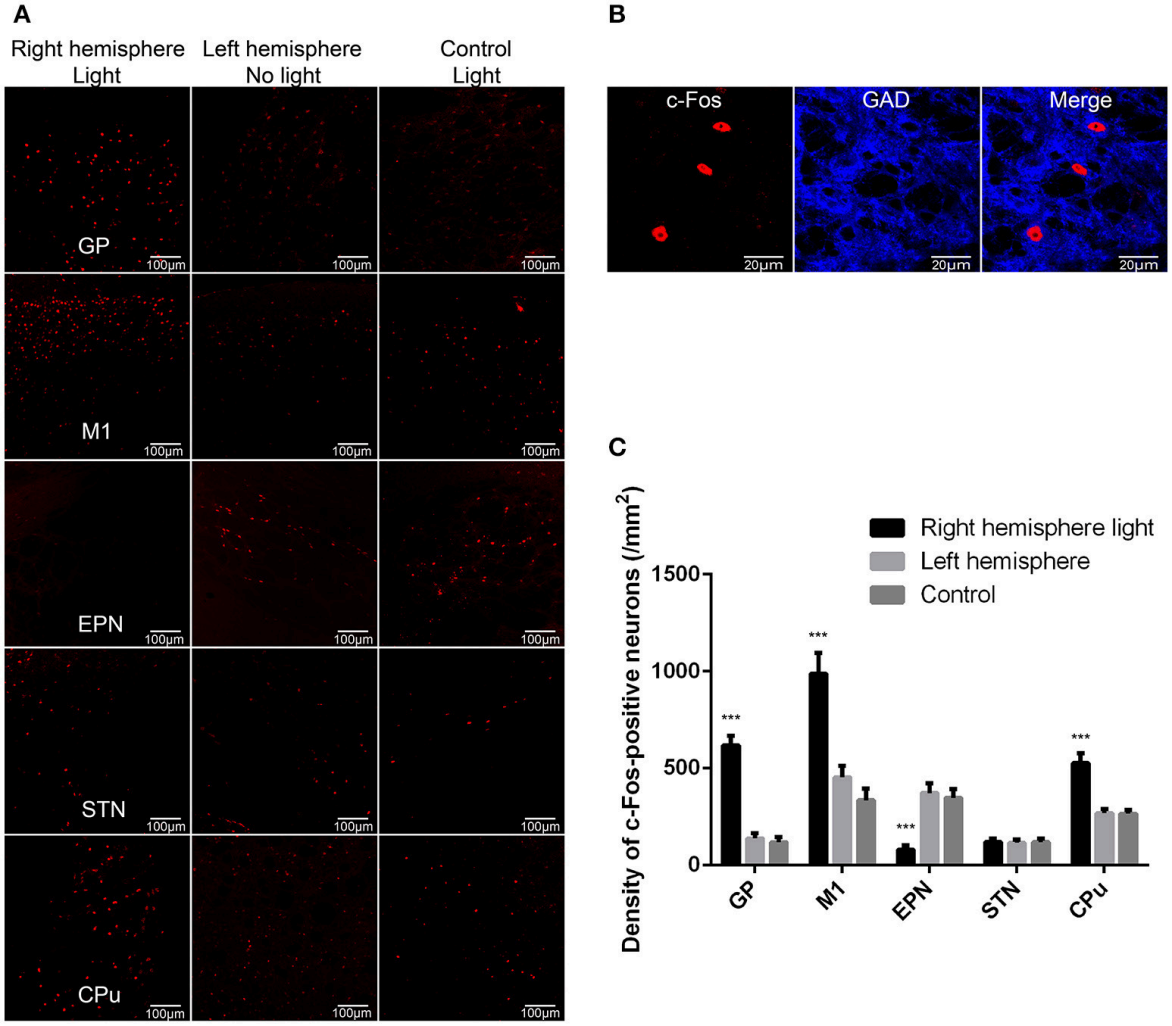
ChR2 stimulation of the GP GABAergic neurons produced the network level changes of basal ganglia circuit as indicated by c-Fos expression in the GP, M1, EPN, STN, and CPu. **(A)** We detected c-Fos immunoreactivity in the light-exposed ipsilateral and contralateral hemispheres of VGAT-ChR2-EYFP transgenic mice, as well as in the light-exposed hemisphere of wild-type mice (control). **(B)** The c-Fos-positive neurons in the GP were all GAD67-positive neurons. **(C)** A bar graph of c-Fos-positive cell counts in the GP, M1, EPN, STN, and CPu. Asterisks indicate significant difference between the c-Fos-positive cells in ipsilateral hemispheres and contralateral hemispheres to optical stimulation {*n* = 10, two-way ANOVA followed by *post-hoc* Tukey's test. There was a significant effect of ChR2 stimulation [*F*_(2,18)_ = 20.377, *p* < 0.001], and brain region effect [*F*_(4,36)_ = 54.76, *p* < 0.001]. A ChR2 stimulation–brain region interaction [*F*_(8,72)_ = 28.22, *p* < 0.001] occurred. *Post-hoc* comparison: GP (right hemisphere light vs. left hemisphere): ***p < 0.001; M1 (right hemisphere light vs. left hemisphere): ***p < 0.001; EPN (right hemisphere light vs. left hemisphere): ***p < 0.001; CPu (right hemisphere light vs. left hemisphere): ***p < 0.001}.

Next, we used c-Fos as a marker for neuronal activity to determine the influence of GP GABAergic neuron activation on the basal ganglia network level activities. The right GP was optically stimulated, and c-Fos expression in the GP, EPN, M1, STN, and CPu was determined and compared between the two hemispheres of the VGAT-ChR2-EYFP transgenic mice and the light-exposed hemisphere of wild-type mice (control). The c-Fos-expressing neurons in the GP were all GAD67-positive neurons (Figures 2A,B), indicating that optical stimulation activated GP GABAergic neurons. ChR2 stimulation of right side GP GABAergic neurons increased the c-Fos-positive neurons in the ipsilateral GP, M1 and CPu, and decreased the c-Fos-positive neurons in the ipsilateral EPN, compared to the contralateral side. There was no statistically significant difference between the c-Fos-positive neurons in the ipsilateral STN and contralateral side (*n* = 10, two-way ANOVA followed by *post-hoc* Tukey's test). There was a significant effect of ChR2 stimulation [*F*_(2,18)_ = 20.377, *p* < 0.001] and brain region effect [*F*_(4,36)_ = 54.76, *p* < 0.001]. A ChR2 stimulation–brain region interaction [*F*_(8,72)_ = 28.22, *p* < 0.001] occurred. *Post-hoc* comparison: GP (right hemisphere light vs. left hemisphere): ^***^p < 0.001; M1 (right hemisphere light vs. left hemisphere): ^***^p < 0.001; EPN (right hemisphere light vs. left hemisphere): ^***^p < 0.001; CPu (right hemisphere light vs. left hemisphere): ^***^p < 0.001; STN (right hemisphere light vs. left hemisphere): *P* = 0.9980 (Figure 2C). These results indicated that excessive GP activity reduced EPN activity and increased M1 and CPu activity.

### ChR2 stimulation of glutamatergic afferent fibers from the STN in the GP produced similar abnormal movements

**Figure 3:**
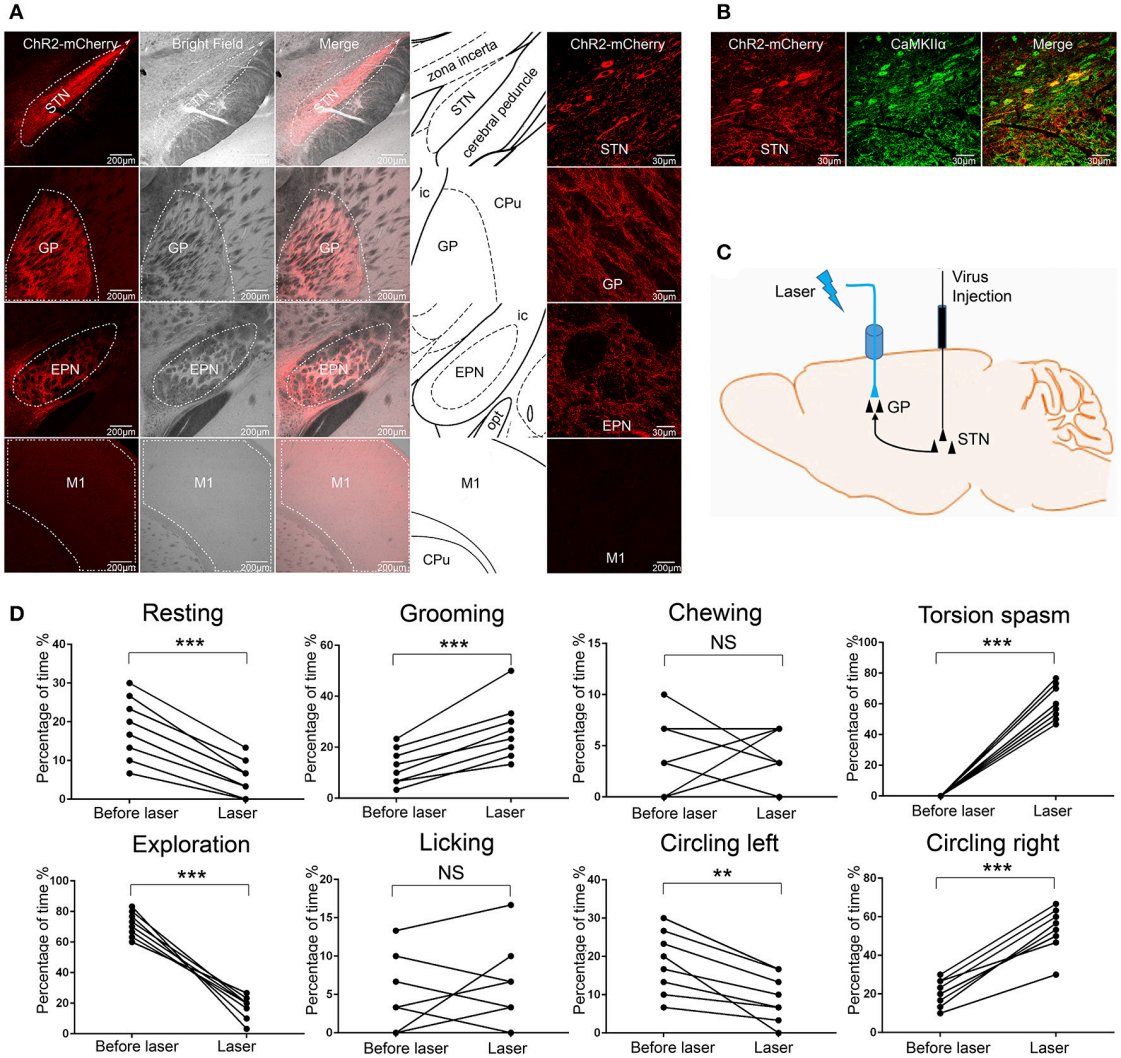
ChR2 stimulation of glutamatergic afferent fibers from the STN in the GP produced similar abnormal movements. **(A)** ChR2-mCherry was selectively expressed in the STN but not in other nearby regions. ChR2-mCherry was also expressed in the two other remote brain regions: GP and EPN, but not in the M1. The selected schematics were adapted from the mouse brain in Stereotaxic Coordinates (Paxinos and Franklin, 2001). **(B)** ChR2-mCherry-expressing STN neurons co-stained with the excitatory neuron-specific marker CaMKIIα. **(C)** Schematic of the optical stimulation of glutamatergic afferent fibers from the STN in the GP. **(D)** The percentage of the 30-s period the animal spent engaging in each behavior. During optical stimulation of the right GP, the mice displayed more grooming, dystonia-like behaviors (torsion of the neck and left forelimb) and circling right, and displayed less resting and exploration [**p < 0.01, ***p < 0.001, *n* = 8, using a paired, two-tailed *t-*test; grooming: *t*_(7)_ = −6.859, *p* < 0.001; torsion spasm: *t*_(7)_ = 15.317, *p* < 0.001; resting: *t*_(7)_ = 8.793, *p* < 0.001; exploration: *t*_(7)_ = 10.604, *p* < 0.001; circling left: *t*_(7)_ = 4.660, *p* = 0.002; circling right: *t*_(7)_ = −10.817, *p* < 0.001; chewing: *t*_(7)_ = −0.261, *p* = 0.802; licking: *t*_(7)_ = −0.475, *p* = 0.649].

The GP primarily receives glutamatergic afferent fibers from the STN (Kita, 2007). To assess the effect of stimulating glutamatergic afferent fibers from the STN in the GP, we injected AAV-CaMKIIα-ChR2-mCherry virus into the right STN. We confirmed that ChR2-mCherry was selectively expressed in the STN but not in other nearby regions (Figure 3A). We also found that ChR2-mCherry was expressed in the two other remote brain regions: the ipsilateral GP and EPN, but not in the ipsilateral M1 (Figure 3A). The selected schematics were adapted from the mouse brain in Stereotaxic Coordinates (Paxinos and Franklin, 2001). The results were consistent with the notion that the GP and EPN are the targets of STN, except for M1 (Hamani et al., 2004). The ChR2-mCherry expression STN neurons were all CaMKIIα positive (Figure 3B). We verified that ChR2 was expressed in the glutamatergic afferent fibers from the STN in the GP. According to current knowledge, one of the distinct advantages of the optogenetic approach is the ability to selectively manipulate basal ganglia projections to define its role in the control of motor and motivation and emotional behavior (Deisseroth, 2014). We implanted the fiber guide cannula above the right GP to selectively stimulate the glutamatergic afferent fibers from the STN in the GP (Figure 3C). Following ChR2 stimulation of the STN axons in the GP (but not the EPN), the mice displayed more stereotyped movements (grooming) and dystonia-like behaviors (torsion of the neck and left forelimb), and displayed less resting and exploration, similar to the behaviors produced by ChR2 stimulation of GABAergic neurons in the right GP in the VGAT-ChR2-EYFP transgenic mice. The mice did not display chewing and licking. The mice rotated in the opposite direction, compared to the VGAT-ChR2-EYFP transgenic mice [^**^p < 0.01, ^***^p < 0.001, *n* = 8, using a paired, two-tailed *t-*test; grooming: *t*_(7)_ = −6.859, *p* < 0.001; torsion spasm: *t*_(7)_ = −15.317, *p* < 0.001; resting: *t*_(7)_ = 8.793, *p* < 0.001; exploration: *t*_(7)_ = 10.604, *p* < 0.001; circling left: *t*_(7)_ = 4.660, *p* = 0.002; circling right: *t*_(7)_ = −10.817, *p* < 0.001; chewing: *t*_(7)_ = −0.261, *p* = 0.802; licking: *t*_(7)_ = −0.475, *p* = 0.649; Figure 3D].

### ChR2 stimulation of glutamatergic afferent fibers from the STN in the GP produced similar network level changes of basal ganglia circuit as indicated by c-Fos expression in the GP, M1, STN, EPN, and CPu

**Figure 4:**
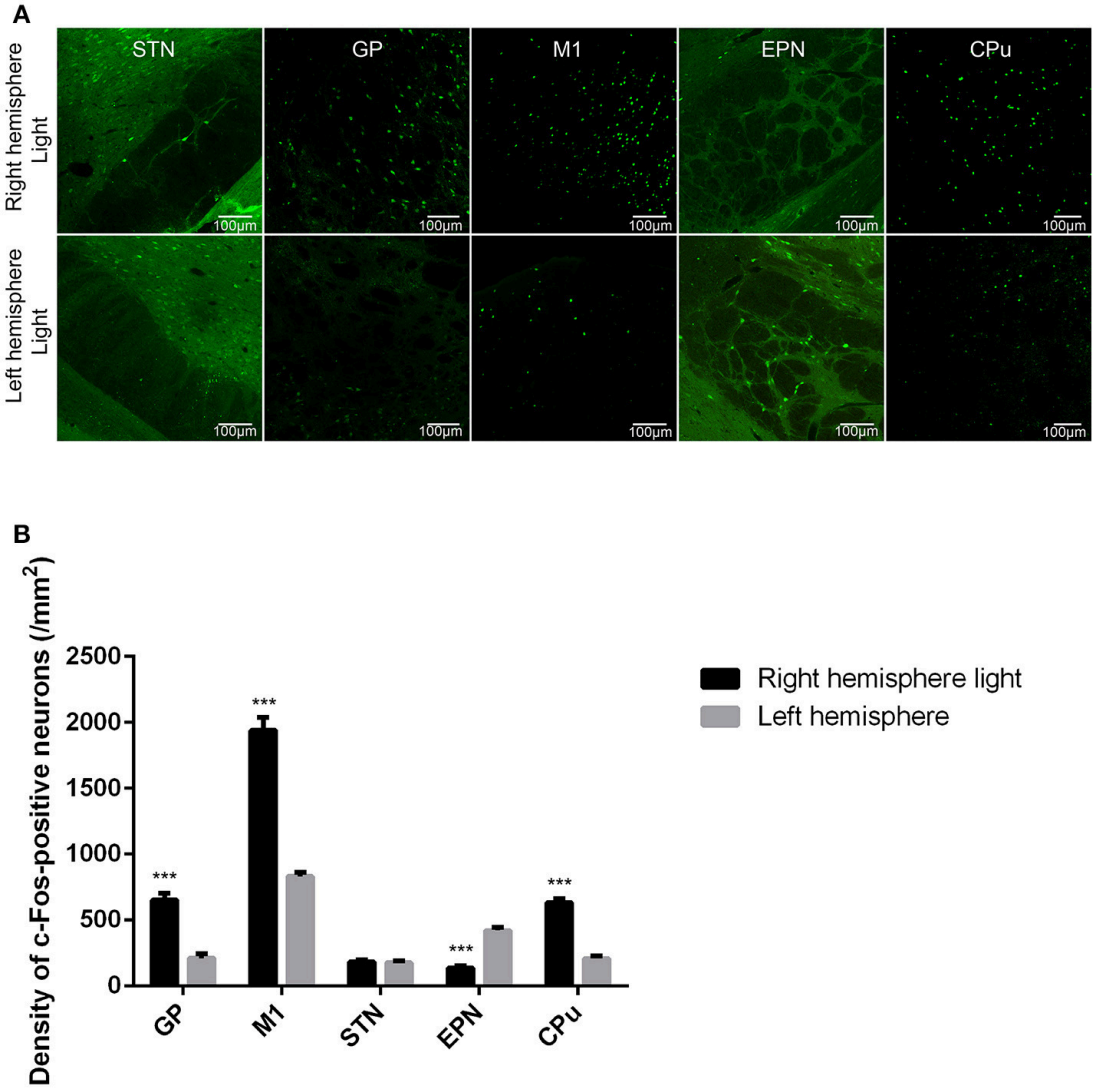
ChR2 stimulation of glutamatergic afferent fibers from the STN in the GP produced similar changes in c-Fos expression in the GP, M1, STN, EPN, and CPu as ChR2 stimulation of GABAergic neurons in the GP. **(A)** c-Fos expression in the light-exposed (right) and contralateral (left) hemisphere of the mouse after ChR2 stimulation of glutamatergic afferent fibers from the STN in the GP. c-Fos expression was detected in the GP, M1, STN, EPN, and CPu. **(B)** Quantitative analysis of c-Fos-positive cell counts in the GP, M1, STN, EPN, and CPu are presented in a bar graph. Asterisks indicate significant differences between the ipsilateral hemisphere and contralateral hemisphere to optical stimulation [***p < 0.001; *n* = 8, using a paired, two-tailed *t-*test; GP: *t*_(7)_ = 11.143, *p* < 0.001; M1: *t*_(7)_ = 14.697, *p* < 0.001; CPu: *t*_(7)_ = 13.353, *p* < 0.001; EPN: *t*_(7)_ = −16.253, *p* < 0.001; STN: *t*_(7)_ = 0.607, *p* = 0.563].

We further investigated the effect of ChR2 stimulation of glutamatergic afferent fibers from the STN in the GP on the expression of c-Fos in the GP, M1, STN, EPN, and CPu. We found that ChR2 stimulation of glutamatergic afferent fibers from the STN in the GP increased the c-Fos-positive neurons in the ipsilateral GP, M1, and CPu, and decreased the c-Fos-positive neurons in the ipsilateral EPN, compared to the contralateral side, similar to the ChR2 stimulation of GP GABAergic neurons in the VGAT-ChR2-EYFP transgenic mice. There was no statistically significant difference between the c-Fos-positive neurons in the ipsilateral STN and contralateral side [^***^p < 0.001; *n* = 8, using a paired, two-tailed *t-*test; GP: *t*_(7)_ = 11.143, *p* < 0.001; M1: *t*_(7)_ = 14.697, *p* < 0.001; CPu: *t*_(7)_ = 13.353, *p* < 0.001; EPN: *t*_(7)_ = −16.253, *p* < 0.001; STN: *t*_(7)_ = 0.607, *p* = 0.563; Figure 4].

## Discussion

There are few studies directly addressing the effect of optogenetic stimulation of GP neurons on motor activity. Here we used the recently developed optogenetics for manipulating the intrinsic GABAergic neurons in GP and glutamatergic afferents from STN in freely moving mice to study the exact role of GP neurons in the control of movement. We found that the light activation of ChR2-expressing GP neurons in the VGAT-ChR2-EYFP transgenic mice produced dystonia-like behaviors (e.g., torsion spasm of the neck and abnormal forelimb posture) and stereotyped movements (repeated grooming, chewing, and licking). This is due to the fact that all ChR2-expressing GABAergic neurons that have an axon passing through or projecting into the GP could be activated in this way. Therefore, it is not quite certain whether the dystonia-like behaviors were caused by excessive activity of the intrinsic GABAergic neurons in the GP. Then, we indirectly drove the firing of GP neurons through activation of their excitatory inputs from the STN by injection of AAV virus containing ChR2 in STN and light stimulation in the GP. Following ChR2 stimulation of the STN axons in the GP (but not the EPN), the mice displayed stereotyped movements (grooming) and dystonia-like behaviors (torsion of the neck and left forelimb) similar to the behaviors produced by ChR2 stimulation of GABAergic neurons in the right GP in the VGAT-ChR2-EYFP transgenic mice. We also noticed that the mice did not display chewing and licking and rotated in the opposite direction compared to the VGAT-ChR2-EYFP transgenic mice. We considered that the dystonia-like behaviors could be mainly caused by excessive activity of the intrinsic GABAergic neurons in the GP. As we know, stimulation of STN axons in the GP mainly drove GP neurons, but the effects of antidromic stimulation also needed to be taken into account. Stimulation of STN axons in the GP likely drives all the other basal ganglia synaptic targets of STN neurons. It was possible that the different behaviors in the two experimental conditions were caused by the wide-spread excitatory effects evoked by STN stimulation. Our finding of the induction of abnormal involuntary movements by optogenetic activation of GABAergic GP neurons collaborates with previous pharmacological, lesioning, and electrophysiology studies (Grabli et al., 2004; Reiner, 2004; Starr et al., 2008), providing new support for the critical role of the GP in the control of motor activity. For example, in primates, local microinjection of the GABAA antagonist bicuculline into the GP induced abnormal involuntary movements (Grabli et al., 2004), while lesioning of the GP with quinolinic acid decreased spontaneous movement (Ayalon et al., 2004). To the best of our knowledge, the present study is the first time to selectively manipulate GABAergic GP neurons in freely moving animals to investigate the critical role of the GP in the control of motor activity.

Additionally, the expression patterns of c-Fos (a marker of neuronal activation) (Sagar et al., 1988) in the five basal ganglia structures (GP, M1, STN, EPN, and CPu) in response to ChR2 activation of the GABAergic GP neurons suggest that the neural circuitry underlying the abnormal involuntary movements is associated with excessive GP, M1, CPu activity and reduced EPN activity. GP GABAergic neurons can be divided into two classes based on their firing patterns, which are termed as “prototypical” neurons and “arkypallidal” neurons. The prototypical and arkypallidal neurons project to distinct targets. Arkypallidal neurons are the major GP cell type input to the striatum, while almost all prototypical neurons innervate STN (Abdi et al., 2015). The activation of arkypallidal neurons was supposed to decrease the c-Fos expression in the striatum. However, in our study we found that the c-Fos-positive neurons in the ipsilateral CPu were increased after the ChR2 stimulation of GP GABAergic neurons. We had to take into account the activation of prototypical neurons. The activation of prototypical neurons will inhibit the EPN through an indirect motor pathway (GP—STN—EPN). We did find the predominant decrease of the c-Fos-positive neurons in the ENP. The ENP is the major inhibitory output nuclei of the BG. The decreased ENP activity will lead to increased cortex activity. Then the increased excitatory input to the CPu from the cortex will lead to increased CPu activity, aligned with the findings in our study. So the reason for c-Fos increases in the CPu could be related to the activation of prototypical neurons in our study. Then, we inferred that prototypical neurons could be more influential in obtaining our results. Recently, Glajch showed that specific activation of arkypallidal neurons suppressed movement (Glajch et al., 2016). Our results are consistent with Glajch's study. Since the arkypallidal neurons suppress movement, the prototypical neurons possibly play different and even opposing roles in motor control, producing hyperkinesia, as shown in our study. This finding is needed to study the exact role of prototypical neurons in movement control in the future.

Another noteworthy observation is that no significant activity decrease was observed in the STN (the major output target of the GP) in response to ChR2 activation of the GABAergic GP neurons. The most likely reason for this is the complex organization of the STN. The STN is also the target of the cortex, thalamus and brainstem, not only the target of the GP (Hamani et al., 2004). We need to take into account the excitatory input to the STN from the cortex. The relationship between STN activity and motor symptoms is complicated. It is reported that both optogenetic excitation and inhibition of STN neurons did not change the PD rats' motor symptoms (Gradinaru et al., 2009). Meanwhile, we found that the c-Fos expression pattern in the GP, M1, STN, EPN, and CPu produced by optogenetic activation of glutamatergic afferent fibers from the STN in the GP was similar to the c-Fos expression pattern produced by optogenetic activation of GABAergic neurons in the GP. Here, although the STN processes were stimulated directly, the c-Fos-positive neurons in the STN did not increase. The most likely reason for this is that STN activity could be affected by other afferent projections, such as GABAergic afferent projections from the GP. These findings support that optogenetic activation of GABAergic neurons in the GP and glutamatergic afferent fibers from the STN in the GP produced similar motor behaviors, which were caused by similar network changes of the basal ganglia circuit. The exact role of the STN in the basal ganglia needs for study in future experiments. Of course, the c-Fos expression is not directly related to neuronal activity. It is still not clear whether the stimulation increased firing rate of GP neurons or promoted synchronization within the GP. In the future, quantifying the light stimulation effect on the electrical activity of GP neurons will be more accurate.

Recently, optogenetic stimulation of intrinsic GP neurons has been shown to increase total sleep by 66% (rather than arousal or motor activity; Qiu et al., 2016), a finding consistent with the early report that lesioning of the GP by kainate produces a huge (45%) increase in arousal (Qiu et al., 2010). The different GP neuronal populations stimulated by ChR2 in Qiu's study (i.e., GABAergic and ChAT neurons in the GP) and ours (GABAergic neurons only under control of VGAT promoter) may account for this difference. The current study failed to address distinct effects of different types of GP GABAergic neurons with distinct molecular identities, firing patterns and complex connections, with likely distinct behavioral responses. However, we speculate that the prototypical neuronal population is the most probable GP GABAergic neuronal population to cause our results. Future studies are needed to address the specific contribution of prototypical GP GABAergic neurons to motor control.

The hyperkinesia that characterizes Huntington Disease (HD) (Ross et al., 2014) is postulated to be related to preferential loss of the indirect pathway neurons that project to the GP (Albin et al., 1990). Because the indirect pathway neurons are GABAergic neurons, this pathology may result in excessive GP activity (GP disinhibition), which could be the neural substrate of HD. Previous studies have also suggested that a reduction in the striatal-GP activity and an increase in the EPN inhibition mediated by GP efferent contribute to dystonia (Hantraye et al., 1990). Also, the neuropathological basis of Tourette's syndrome (TS) has been attributed to the disruption of local inhibitory circuits within the striatum-GP circuit, which would lead to the aberrant activation of the cortical-basal ganglia loop, resulting in abnormal tic-like movements (Albin and Mink, 2006; Felling and Singer, 2011; Bronfeld and Bar-Gad, 2013). Our findings that optogenetic stimulation of the intrinsic GP GABAergic neurons or the STN-GP glutamatergic projections in the GP produced abnormal movements support the view that excessive GP activity could be the neural substrate of the abnormal involuntary movements found in movement disorders such as Huntington's disease and dystonia. Because the prototypical neuron population is the most probable GP GABAergic neuronal population to cause our results, we speculate that excessive prototypical neurons activity could be the neural substrate of the abnormal involuntary movements. Inhibition of GP GABAergic neurons, mainly prototypical neurons through surgical means, deep brain stimulation (DBS) or drug-based interventions, represent new treatment targets for hyperkinetic movement disorder. Of high importance is to understand the functional role of different types of GP neurons in the basal ganglia. Certainly, there is much more to be discovered.

## Conclusion

To our knowledge, there are few studies directly addressing the effect of optogenetic stimulation of GP neurons on motor activity. Here, we found that optogenetic stimulation of GABAergic neurons in the GP or glutamatergic afferent fibers from the STN in the GP, produced hyperkinesia. Our data supported the important role of the GABAergic GP neurons, mainly prototypical neurons, in the control of movement. Albeit, there are still a lot of questions ahead of us. Future studies are needed to address the specific contribution of prototypical GABAeregic GP neurons to motor control.

## Author contributions

JT designed and performed the study. JT and JC contributed to the major writing of manuscript. YY analyzed the data. WX was engaged in EEG recordings. RZ provided help in mice surgery. HL did some mice behavioral tests. All authors have reviewed and edited the manuscript. SD, JC, and BZ supervised the study. BZ is designated correspondence on the manuscript.

### Conflict of interest statement

The authors declare that the research was conducted in the absence of any commercial or financial relationships that could be construed as a potential conflict of interest.
